# The psychological impact of working in an oncology center

**DOI:** 10.1192/j.eurpsy.2023.1839

**Published:** 2023-07-19

**Authors:** N. Khouja, A. Koubaa, J. Hsinet, E. Baraketi, S. Ismail, G. Trabelsi, A. Benzarti, A. Ben Jemaa

**Affiliations:** 1 Service de la Médecine du travail et des Maladies professionnelles, Centre hospitalo-universitaire La Rabta, Tunis, Tunisia

## Abstract

**Introduction:**

Paramedical staffs in oncology are among the most exposed to stress factors in the workplace, predisposing them to develop certain psychiatric pathologies, in particular anxiety disorders, depressive syndromes and burnout.

**Objectives:**

The objective of this study was to study these psychosocial risk factors and to detect the psychological repercussions on this professional category.

**Methods:**

Our cross-sectional, mono-centric, descriptive and analytical study was conducted between November 1^st^ and 8^th^, 2022 at the Salah Azaiez Institute in Tunis. The screening of anxiety and depression was performed with the Hospital Anxiety and Depression Scale (HADS), the assessment of Burnout was performed with the Maslach Burnout Inventory (MBI).

**Results:**

Fifty-four workers were included (mean age 39.72 years and sex ratio 0.22). According to the HADS scale, 68.5% had definite or doubtful anxiety symptoms, 51.8% had definite or doubtful depression symptoms. According to the Maslach scale, 59.3% had a high burnout score, 37% had a high depersonalization score and 38.9% had a low personal accomplishment score. A combination of all three was present in 7.4% of the staff. Anxiety was associated with workplace violence and lack of career prospects, depression was associated with lack of leisure activities, and burnout was associated with age, emergency management for the emotional exhaustion score, and the number of children, workload and workplace violence.

**Conclusions:**

Working in an oncology environment seems to be associated with a number of factors that could significantly increase the risk of psychiatric pathology. Some of these factors are perfectly modifiable, which opens up prospects for targeted preventive actions.

**Disclosure of Interest:**

None Declared

